# Exploring public interest in gut microbiome dysbiosis, NAFLD, and probiotics using Google Trends

**DOI:** 10.1038/s41598-023-50190-5

**Published:** 2024-01-08

**Authors:** Salvatore Pezzino, Maria Sofia, Chiara Mazzone, Giorgia Litrico, Marcello Agosta, Gaetano La Greca, Saverio Latteri

**Affiliations:** Department of Surgical Sciences and Advanced Technologies “G. F. Ingrassia”, Cannizzaro Hospital, University of Catania, Catania, Italy

**Keywords:** Microbiota, Liver diseases

## Abstract

Scientific interest related to the role of gut microbiome dysbiosis in the pathogenesis of non-alcoholic fatty liver disease (NAFLD) has now been established and is constantly growing. Therefore, balancing dysbiosis, through probiotics, would be a potential therapy. In addition to scientific interest, on the other hand, it is important to evaluate the interest in these topics among the population. This study aimed to analyze, temporally and geographically, the public's interest in gut microbiome dysbiosis, NAFLD, and the use of gut probiotics. The most widely used free tool for analyzing online behavior is Google Trends. Using Google Trends data, we have analyzed worldwide volume searches for the terms “gut microbiome”, “dysbiosis”, “NAFLD” and “gut probiotic” for the period from 1, January 2007 to 31 December 2022. Google's relative search volume (RSV) was collected for all terms and analyzed temporally and geographically. The RSV for the term “gut microbiome” has a growth rate of more than 1400% followed, by “gut probiotics” (829%), NAFLD (795%), and “dysbiosis” (267%) from 2007 to 2012. In Australia and New Zealand, we found the highest RSV score for the term “dysbiosis” and “gut probiotics”. Moreover, we found the highest RSV score for the term “NAFLD” in the three countries: South Korea, Singapore, and the Philippines. Google Trends analysis showed that people all over the world are interested in and aware of gut microbiome dysbiosis, NAFLD, and the use of gut probiotics. These data change over time and have a geographical distribution that could reflect the epidemiological worldwide condition of NAFLD and the state of the probiotic market.

## Introduction

The human digestive system is hosted to between 500 and 1000 types of commensal bacteria, the great majority of which are anaerobic^1,2^. The gut microbiome’s dysbiosis has been associated with several disorders, including chronic inflammation^3^, neurological and ophthalmic diseases^4,5^, and chronic liver diseases such as non-alcoholic fatty liver disease (NAFLD), a condition caused by an excessive accumulation of fat in hepatocytes^6–8^. By 2030, the rate of increase in NAFLD incidence is expected to be quadruplicated^9,10^. The epidemic rise of NAFLD can be attributed in large part to the modern plagues of sedentary living, high-calorie diets, and low levels of physical activity^11,12^. One of the etiologies of NAFLD has been linked to dysbiosis in the gut microbiome. There is mounting evidence from both preclinical and clinical studies demonstrating that gut dysbiosis is a significant element in the development of this dysfunction and that this disturbance plays a substantial role in NAFLD pathogenesis^6–8,13–17^.

A probiotic is “live bacteria that, when administered in suitable proportions, provide health benefits to the host”^18^. Studies on the efficacy of probiotics in reversing dysbiosis have shown encouraging results^19–21^. In a number of studies, both preclinical and clinical, probiotics have shown promise in correcting gut dysbiosis and clinical markers of NAFLD illness^22–27^.

Scientific interest related to the role of gut microbiome dysbiosis in the pathogenesis of NAFLD has now been established and is constantly growing^6,14,17^. Therefore, balancing dysbiosis, through probiotics, would be a potential therapy^20,21^. In addition to scientific interest, on the other hand, it is important to evaluate the interest in these topics among the population. The most widely used free tool for analyzing online behavior is Google Trends, which can also give real-time data about trends and changes in online interest over time for different terms and themes^28–30^. Electronic data is utilized in this field to monitor online interest in health problems and procedures, predict disease outbreaks, and assess the efficacy of health education efforts^31^. For example, in a recent study, Mikoła et al. showed how interest in probiotic-related information may be correlated with antibiotic consumption, health expenditures, and the country's level of development^32,33^.

The purpose of our study is to analyze, temporally and geographically, the public's interest related to gut microbiome dysbiosis, NAFLD, and the use of gut probiotics using Google Trends data, so that physicians and researchers should not only be aware of the hotspots of scientific research but also of the concerns of public interest.

## Results

### Relative search volume (RSV) trend

**Figure 1:**
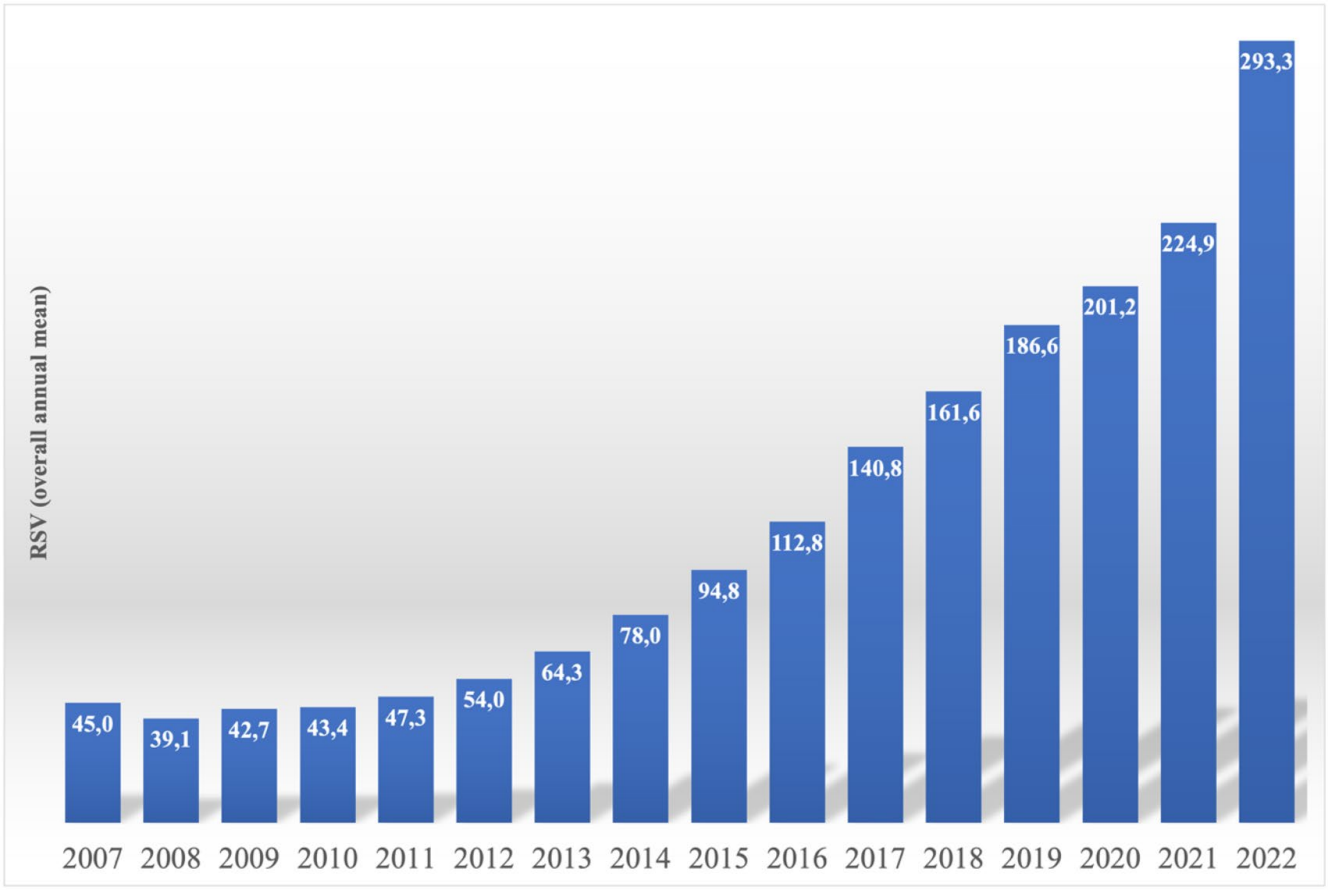
World annual mean trend of the RSV (relative search volume) from January 2007 until December 2022 (15 years), given by the sum of the mean RSV value of each searched term from Google Trends, data accessed on January 18, 2023.

Figure 1 depicts the overall global RSV trends for all four searched terms from 2007 to 2022. RSV grew from 45 to about 293. There was a small negative decline between 2008 and 2012. Despite minor fluctuations, the average search strength demonstrated an overall upward tendency. The curve started to rise again in 2013.

**Figure 2:**
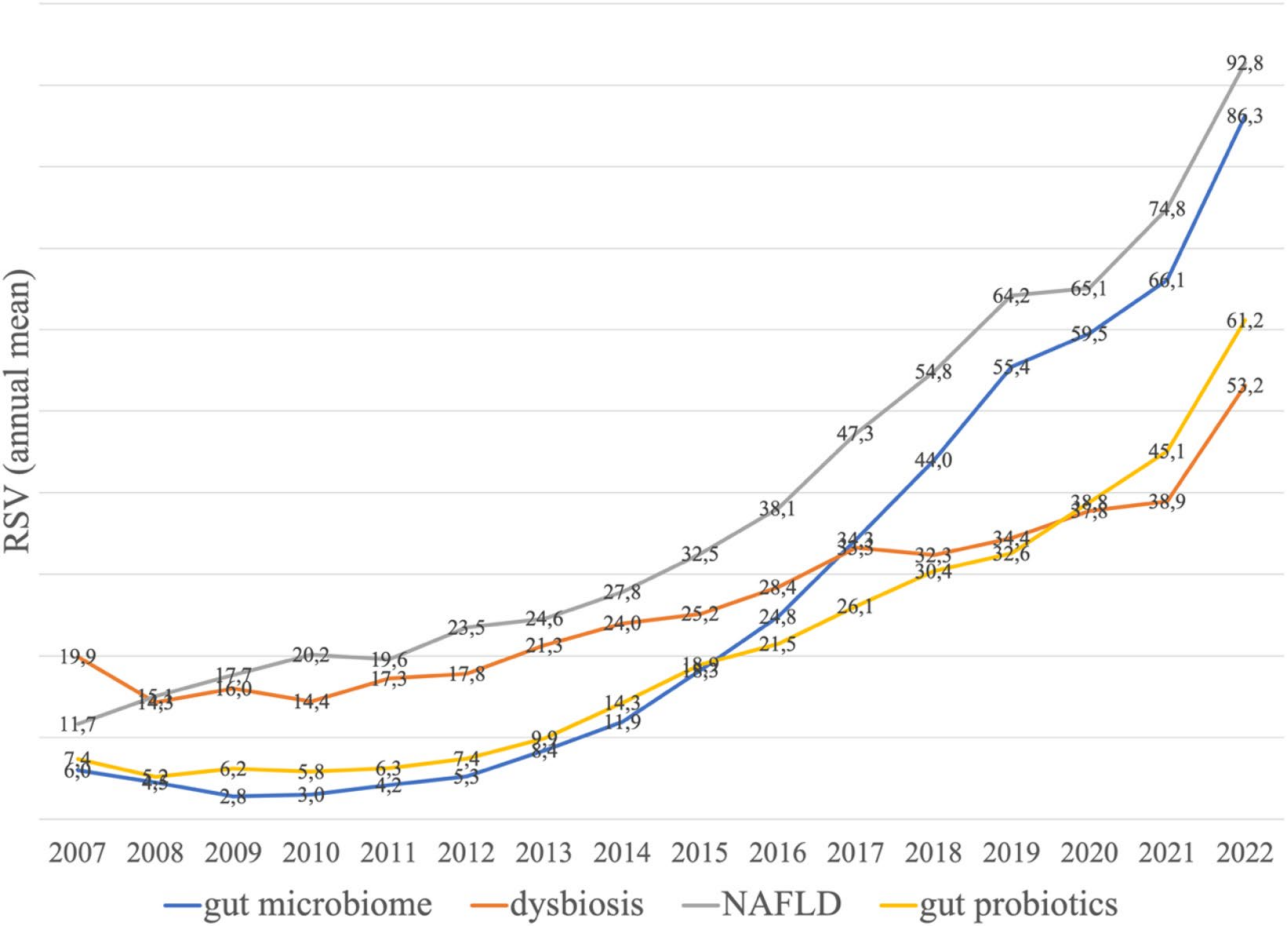
World annual mean trend of the RSV (relative search volume) from January 2007 until December 2022 (15 years) for every single searched term: “gut microbiome”, “dysbiosis”, “NAFLD” and “gut probiotics” from Google Trends. RSV: relative search volumes; data accessed on January 18, 2023.

Among the four searched terms, “gut microbiome” and “gut probiotics”, changed respectively from an RSV of 6 and 7.4 to an RSV of 86.3 and 61.2. While “dysbiosis” and “NAFLD" have respectively an increase from 19.9 and 11.7 to 53.2 and 92.8 (Fig. 2).

**Figure 3:**
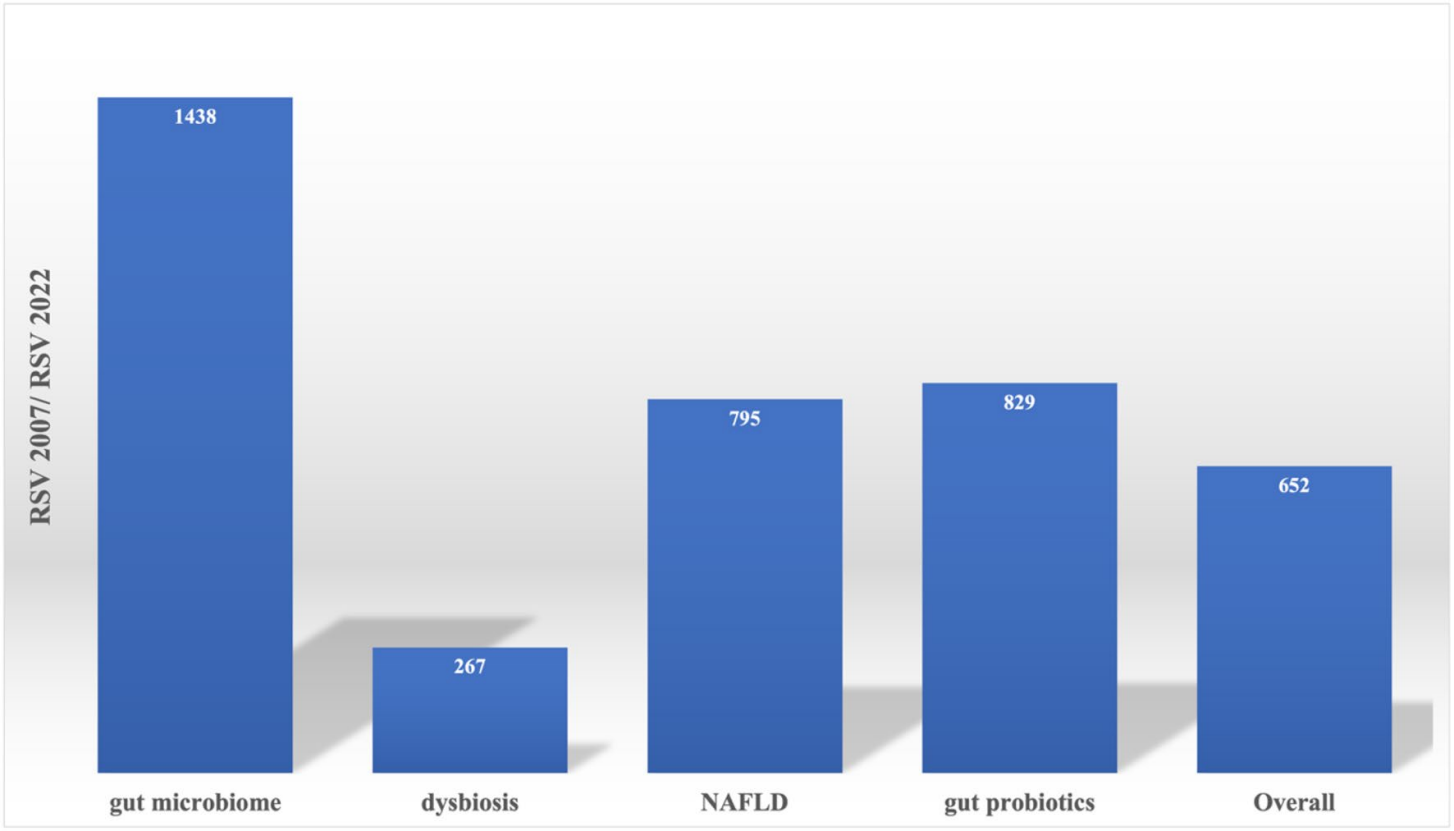
The ratio (%) between the RSV (relative search volume) value of the year 2022 and the value of the year 2007 for the searched terms: “gut microbiome, “dysbiosis”, “NAFLD” and “gut probiotics.

When comparing the RSV value for the year 2022 to the value for the year 2007 for all four words (Fig. 3), the term “gut microbiome” shows a growth of about 1400%, followed by "gut probiotics" (829%), NAFLD (795%), and “dysbiosis” with 267%.

### Country relative search volume (RSV) distribution

**Figure 4:**
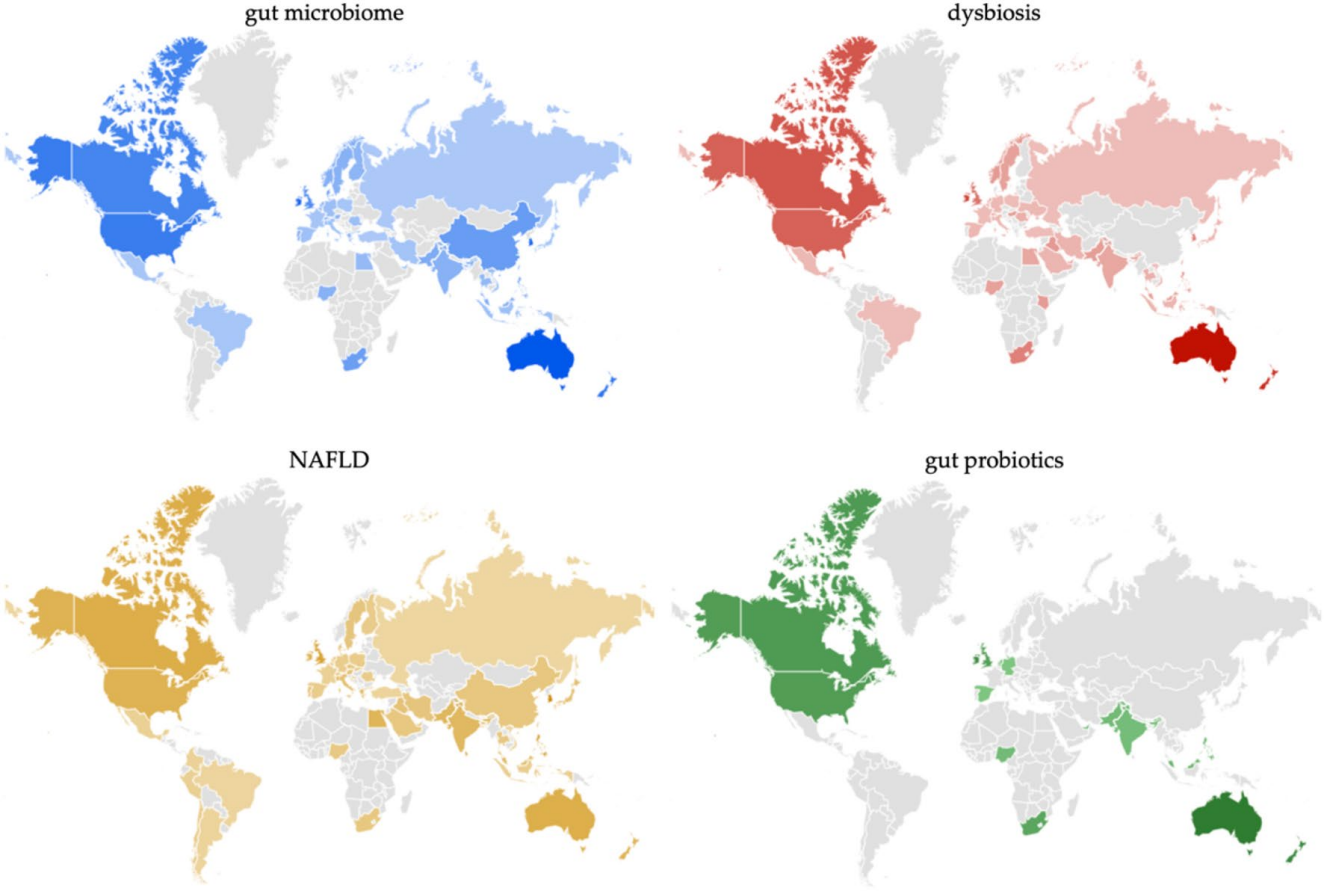
Geographic distribution of the RSV (relative search volume) for the searched terms from Google Trends: “gut microbiome” is colored in blue, “dysbiosis” is colored in red, "NAFLD" is colored in yellow, and “gut probiotics” is colored in green. The color gradient is proportional to the value of RSV; data accessed on January 18, 2023.

The geographic distribution graph for each searched term from 2007 to 2022 is shown in Fig. 4, while Table 1 shows the country RSV value for searched terms.

**Table 1:** Country RSV (relative search volume) value for searched terms: “gut microbiome”, “dysbiosis”, “NAFLD,” and “gut probiotics” from Google Trends.

Country	gut microbiome (RSV)	Country	dysbiosis (RSV)	Country	NAFLD (RSV)	Country	gut probiotics (RSV)
Australia	100	Australia	100	Argentina	6	Australia	100
Austria	19	Belgium	12	Australia	55	Canada	60
Bangladesh	27	Brazil	4	Austria	28	Germany	3
Belgium	19	Canada	61	Bangladesh	50	India	14
Brazil	6	Egypt	16	Belgium	24	Ireland	78
Canada	61	France	5	Brazil	5	Malaysia	24
China	41	Germany	6	Canada	56	Netherlands	8
Denmark	37	Hong Kong	46	Chile	15	New Zealand	81
Egypt	16	Hungary	19	China	23	Nigeria	16
Finland	20	India	16	Colombia	8	Pakistan	16
France	7	Indonesia	8	Croatia	19	Philippines	23
Germany	12	Iran	9	Czechia	22	Singapore	68
Greece	16	Iraq	23	Denmark	25	South Africa	44
Hong Kong	71	Ireland	70	Egypt	46	Spain	2
India	23	Italy	6	Finland	16	United Arab Emirates	26
Indonesia	11	Japan	7	France	10	United Kingdom	52
Iran	8	Kenya	22	Germany	15	United States	57
Ireland	90	Malaysia	18	Greece	36		
Israel	25	Mexico	6	Hong Kong	47		
Italy	9	Netherlands	13	India	41		
Japan	7	New Zealand	82	Indonesia	13		
Malaysia	25	Nigeria	19	Iran	25		
Mexico	6	Norway	23	Iraq	28		
Netherlands	29	Pakistan	21	Ireland	47		
New Zealand	71	Philippines	21	Israel	29		
Nigeria	23	Poland	4	Italy	30		
Norway	25	Romania	11	Japan	32		
Pakistan	28	Russia	4	Malaysia	33		
Philippines	25	Saudi Arabia	8	Mexico	10		
Poland	6	Singapore	35	Netherlands	23		
Portugal	22	South Africa	37	New Zealand	47		
Romania	12	South Korea	36	Nigeria	20		
Russia	4	Spain	7	Pakistan	43		
Singapore	75	Sweden	19	Peru	11		
South Africa	39	Swiss	18	Philippines	73		
South Korea	84	Taiwan	23	Poland	16		
Spain	10	Thailand	11	Portugal	27		
Sweden	23	Turkey	4	Romania	15		
Swiss	27	Ukraine	6	Russia	3		
Taiwan	22	United Arab Emirates	23	Saudi Arabia	22		
Thailand	17	United Kingdom	45	Singapore	73		
Turkey	4	United States	55	Slovakia	26		
United Arab Emirates	20	Vietnam	3	South Africa	22		
United Kingdom	53			South Korea	100		
United States	68			Spain	15		
Vietnam	11			Sri Lanka	51		
				Sweden	25		
				Swiss	34		
				Taiwan	29		
				Thailand	21		
				Turkey	6		
				United Arab Emirates	30		
				United Kingdom	67		
				United States	50		
				Vietnam	5		

RSV was calculated from searches for all four terms in the corresponding country (from January 1, 2007, to December 31, 2022). Only countries with data in at least one of the four terms are included. The data are sorted by country name in descending order; the data were accessed on January 18, 2023.

The top ten countries with the highest RSV for the term “gut microbiome” are Australia (RSV = 100), Ireland (RSV = 90), South Korea (RSV = 84), Singapore (RSV = 75), New Zealand (RSV = 71), Hong Kong (RSV = 71), United States (RSV = 68), Canada (RSV = 61), United Kingdom (RSV = 53), China (RSV = 41).

The top ten RSV countries for the term “dysbiosis” are Australia (RSV = 100), New Zealand (RSV = 82), Ireland (RSV = 70), Canada (RSV = 61), United States (RSV = 55), Hong Kong (RSV = 46), United Kingdom (RSV = 45), South Africa (RSV = 37), South Korea (RSV = 36), Singapore (RSV = 35).

For the term “NAFLD”, the top ten are South Korea (RSV = 100), Singapore (RSV = 73), Philippines (RSV = 73), United Kingdom (RSV = 67), Canada (RSV = 56), Australia (RSV = 55), Sri Lanka (RSV = 51), United States (RSV = 50), Bangladesh (RSV = 50), Hong Kong (RSV = 47), Ireland (RSV = 47), New Zealand (RSV = 47), Egypt (RSV = 46).

The following countries are in the top ten ranks for the term “gut probiotics”: Australia (RSV = 100), New Zealand (RSV = 81), Ireland (RSV = 78), Singapore (RSV = 68), Canada (RSV = 60), United States (RSV = 57), United Kingdom (RSV = 52), South Africa (RSV = 44), United Arab Emirates (RSV = 26), Malaysia (RSV = 24).

On the other hand, concerning countries exhibiting the lowest RSV for the term "gut microbiome," the five ranked in ascending order are Russia (RSV = 4), Turkey (RSV = 4), Brazil (RSV = 6), Mexico (RSV = 6) and Poland (RSV = 6).

Instead, the five countries that rank lowest for the term “dysbiosis” are Vietnam (RSV = 3), Brazil (RSV = 4), Poland (RSV = 4), Russia (RSV = 4) and Turkey (RSV = 4).

For the term “NAFLD” are Russia (RSV = 3), Brazil (RSV = 5), Vietnam (RSV = 5), Argentina (RSV = 6), and Turkey (RSV = 6).

Finally, the five countries with the lowest RSV for the term “gut probiotics” are Spain (RSV = 2), Germany (RSV = 3), the Netherlands (RSV = 8), India (RSV = 14), and Nigeria (RSV = 16).

### Relative search volume (RSV) of associated queries

The top ten queries related to the searched terms from 2007 to 2022 are listed in Table 2.

**Table 2:** The most used related topics for the period 2007–2022, from Google Trends.

gut microbiome RSV		dysbiosis RSV		NAFLD RSV		gut probiotics RSV	
the gut microbiome	100	gut	100	nafld liver	100	probiotics for gut	100
human gut	43	dysbiosis symptoms	25	liver	94	gut health probiotics	87
human microbiome	41	what is dysbiosis	20	nafld nash	62	gut health	86
human gut microbiome	41	microbiota dysbiosis	18	nash	62	probiotics for gut health	49
gut health	35	microbiota	18	nafld score	50	best probiotics	45
gut microbiome bacteria	27	dysbiosis diet	18	fatty liver	46	best gut probiotics	44
gut bacteria	27	microbiome dysbiosis	17	nafld disease	39	probiotic	41
gut microbiota	26	microbiome	17	fibrosis	28	gut bacteria	30
bacteria	26	dysbiosis definition	17	liver disease	27	leaky gut	29
microbiota	26	dysbiosis treatment	16	nafld fibrosis score	20	good probiotics	21
the human microbiome	23	dysbiosis meaning	14	fibrosis score	20	good gut probiotics	20
what is gut microbiome	19	dysbiosis test	12	nafld diet	20	best probiotics for gut health	19
what is microbiome	19	probiotics	10	nafld symptoms	18	probiotics foods	17
microbiome diet	18	gut microbiota	10	fatty liver disease	17	what is gut	15
gut microbiome diet	18	gut microbiota dysbiosis	10	nafld treatment	16	what is probiotics	14
gut microbiome disease	15	dysbiosis of the gut	10	what is nafld	15	best probiotic	14
microbiome test	14	candida	9	cirrhosis	14	gut flora	14
healthy gut	14	bacterial dysbiosis	9	nafld cirrhosis	14	prebiotics	13
healthy gut microbiome	14	sibo	9	nash liver	13	healthy gut	12
what is the gut microbiome	12	leaky gut	8	nafld medical abbreviation	11	probiotics for leaky gut	11
what is the microbiome	12	gut dysbiosis symptoms	8	icd 10 nafld	11	probiotic for gut health	11
gut microbiome foods	12	gut microbiome dysbiosis	7	alt	10	probiotics food	11
gut microbiome brain	11	microbial dysbiosis	7	nafld vs nash	9	probiotics supplements	10
gut microbiome and disease	11	gut microbiome	7	nafld causes	9	microbiome	9
probiotics	10	probiotic	6	steatosis	8	gut microbiome	9

Users who searched for the terms “gut microbiome”, “dysbiosis”, “NAFLD”, and “gut probiotics”, also searched for these topics. The score is on a relative scale: 100 indicates the most searched query, and 50 indicates a query with half the searches compared to the most searched query; RSV = relative search volume; data accessed on January 18, 2023.

Among the top-ten topics related to the term “gut microbiome”, apart from the variation with the article of the search term (the gut microbiome = 100 RSV), we found human gut (RSV = 43), human microbiome (RSV = 41) and human gut microbiome (RSV = 41), gut health (RSV = 35), gut microbiome bacteria (RSV = 27) and gut bacteria (RSV = 27), gut microbiota (RSV = 26), bacteria (RSV = 26) and microbiota (RSV = 26).

Among the most closely connected topics to “dysbiosis” term, we observed: gut (RSV = 100), dysbiosis symptoms (RSV = 25), what is dysbiosis (RSV = 20), microbiota dysbiosis (RSV = 18), microbiota (RSV = 18), dysbiosis diet (RSV = 18), microbiome dysbiosis (RSV = 17), microbiome (RSV = 17), dysbiosis definition (RSV = 17), dysbiosis treatment (RSV = 16).

The top ten results for "NAFLD," are the following: nafld liver (RSV = 100), liver (RSV = 94), nafld nash (RSV = 62), nash (RSV = 62), nafld score (RSV = 50), fatty liver (RSV = 46), nafld disease (RSV = 39), fibrosis (RSV = 28), liver disease (RSV = 27), nafld fibrosis score (RSV = 20), fibrosis score (RSV = 20), nafld diet (RSV = 20).

Top-related topics to "gut probiotics" include probiotics for gut (RSV = 100), gut health probiotics (RSV = 87), gut health (RSV = 86), probiotics for gut health (RSV = 49), best probiotics (RSV = 45), best gut probiotics (RSV = 44), probiotic (RSV = 41), gut bacteria (RSV = 30), leaky gut (RSV = 29), good probiotics (RSV = 21).

## Discussion

Nowadays, open data from the internet is being used more and more in health research. Understanding and monitoring public perceptions of disease management and treatment are equally crucial. Google Trends is rapidly becoming the most popular tool for recognizing internet activity^34^. In our study using Google search traffic data, we observed public interest and knowledge consisting of annual trends, geographic distribution and related topics related to gut microbiome dysbiosis, NAFLD, and the usage of gut probiotics.

RSV annual trend showed an overall increasing trend, although with slight fluctuation, and a decline in RSV from years 2008 to 2012 (Fig. 1). The increase is higher, especially in the last year, with an RSV that varies from a value of 224.9 to a value of 293.3.

Observing the distinct search terms, we could find that those with a considerable increase in RSV values are those related to the terms "gut microbiome", followed by "gut probiotics", “NAFLD” and finally “dysbiosis” (Fig. 2).

Looking at the 2022 to 2007 RSV ratio data (Fig. 3), we found that all search terms follow a significant percentage increase: the term "gut microbiome" has a growth of more than 1 400%, followed by "gut probiotics" (829%), NAFLD (795%), and "dysbiosis" with 267%.

These trends over time reflect the growing research interest in the dysbiosis of the gut microbiome, its role in several diseases including NAFLD and the interest in gut probiotics as a therapy to restore dysbiosis.

In recent years, the demand for probiotic bacteria-containing products has increased. More and more people are adding probiotic bacteria to their diets as functional food supplements because of the positive effects they have on health^35–37^. When it comes to functional food ingredients, probiotics are by far the most popular choice. Studies have shown that probiotic strains can help treat a number of illnesses, including gastrointestinal distress, bacterial vaginosis, and urinary tract infections^38–40^. Protection from dangerous bacteria in the digestive tract, better bowel movement, reduced cholesterol levels, relief from lactose intolerance and inflammation, and so on are just some of the health benefits associated with probiotics^41,42^.

Another interesting finding in this study was the variation in interest in the searched terms across countries (Fig. 4 and Table 1).

Interestingly, in Australia, and New Zealand, we found the highest RSV score for the term “dysbiosis” and “gut probiotics”. Australia is also in the first rank for the term “gut microbiome”.

From 2021 to 2030, the worldwide probiotics industry is anticipated to increase from 2021’s valuation of about USD 58 billion with a CAGR (compound annual growth rate) of 7.5%^43,44^. Rising public interest in preventative medicine and the refinement of highly effective probiotic strains are driving the industry. When taken in sufficient quantities, probiotics have positive benefits on the body, including enhanced gastrointestinal wellness and decreased intestinal inflammation^45,46^. By bolstering the immune system, probiotics play an important role in preventing the onset of illness^45,46^. Hence, increasing focus on preventative medicine is anticipated to fuel market expansion throughout the forecast period. More than 40.0% of worldwide probiotic market revenue was generated in the Asia Pacific area in 2021^43,44^. Increased consumer awareness is largely attributed to the competitive strategies implemented by multinational companies in the region. Market expansion in the region is anticipated to be driven by a number of factors, including a rising population, rising disposable income, and an increasing standard of life^43,44,47^. The association between RSV and the topic of probiotics and dysbiosis might be caused by the recommendations of probiotics to restore the gut microbiome^12,46^.

As far as the “NAFLD” term we found the highest RSV score in the three countries: South Korea, Singapore and the Philippines. An estimated 32.4% of the world's population has NAFLD, and that number has been steadily rising over the past decade^48^**.** Among Asian countries, NAFLD is more recent (27%), compared to Europe and North America (24%)^49^. From 5% in Singapore to 30% in Indonesia, the prevalence of NAFLD in Asia substantially varies^50,51^. The prevalence of metabolic diseases is on the rise in Korea, from 24.9% in 1998 to 29.0% in 2013^52^. This has become a major public health issue^50,51^. In Singapore, the number of people living with NAFLD is expected to increase from 1,492,000 in 2019 to 1,799,000 in 2030^53^. In addition, it is concerning that at least one-third of the general population in the Philippines has NAFLD; the prevalence rate is 38%, compared to 25% globally^54^. It is believed that the rising prevalence of NAFLD in the Asia–Pacific area is due to the widespread adoption of Western food and lifestyle practices and predisposing genetic factors among many different communities^55^. Rapid industrialization in many Asian nations has led to a shift toward sedentary behaviors and overnutrition, both of which are strongly linked to metabolic problems^56^. Moreover, despite the fact that obesity and diabetes are well-known risk factors for the disease^57,58^, a sizable population in the Asian region suffers from NAFLD while having a healthy weight^59^. It is expected that the number of people diagnosed with NAFLD and NASH throughout Asia will continue to rise, especially in rapidly aging populations, who are more likely to develop chronic lifestyle-related illnesses^60^.

The top ten related queries in terms of searches from 2007 to 2022 are listed in Table 2. The queries related to the term "gut microbiome", are related to understanding by the public of what the gut microbiome is, what bacteria it consists of, how to diagnose gut dysbiosis, and how to keep it healthy through diet, food, and also probiotics. In addition, some search terms refer to the link between the gut and brain, the so-called gut-brain axis. The gut-liver axis is increasingly being recognized as the underlying cause of NAFLD^14,16,17^. By dysregulating the gut-liver axis, gut microbiome dysbiosis, which contributes to the development of NAFLD, promotes an increase in intestinal permeability and uncontrolled transport of microbial metabolites into the liver^14,16,17^. Moreover, NAFLD is facilitated by a dysfunctional gut-liver axis, which affects hepatocyte lipid and glucose metabolism and disturbs the equilibrium of inflammatory mediators^14,16,17^.

The public queries related to the term "dysbiosis" are related to its definition, the pathogenetic mechanisms that underlie it, possible diagnosis, and treatment.

The queries related to the term "NAFLD" are related to its definition and pathogenetic mechanisms, the correlation with non-alcoholic steatohepatitis (NASH), the diagnosis of fibrosis, and care through diet. Research has connected dysbiosis in the gut microbiome not only to the development of NAFLD but also to its progression in NASH, which is characterized histologically by hepatocyte damage and various degrees of fibrosis, up until cirrhosis^6–8,13^.

The queries related to the terms “gut probiotics” are related to the public's search for an "effective" probiotic treatment. Among related queries to “gut probiotics” appear also the terms “leaky” or “probiotics for the leaky gut”. The intestinal barrier permits communication between the digestive tract and the liver^61,62^. Tight connections between intestinal cells are essential for maintaining the integrity of the intestinal barrier^63^. A leaky gut, defined by decreased intestinal barrier function, is a well-established characteristic of dysbiosis in NAFLD patients^64,65^. Dysbiosis and associated reduced gut permeability results in an increase in gut-derived toxins in the systemic circulation, constituting metabolic endotoxemia, which contributes to the development of the persistent low-grade inflammation observed in NAFLD^65,66^.

In conclusion, Google Trends analysis showed that people all over the world are interested in and aware of gut microbiome dysbiosis, NAFLD, and the use of gut probiotics. These data change over time and have a geographical distribution that could reflect the epidemiological worldwide condition of NAFLD and the state of the probiotic market. The collection of such information has the potential to improve the monitoring of numerous diseases in the future. A number of variables limit Google Trends' applicability. Because Google Trends removes queries from the same person over a short time period to reduce counts of continued searching, the service may suffer from sampling bias. Google's search algorithms are constantly being improved, so the same query terms may provide different results. This will make it more challenging to replicate these studies, which rely on reproducible results. Whether or not people learn more about health issues as a result of using Google to research them is debatable. This is because Google Trends only reports how often a term has been searched without providing any information on the quality of the results that may be used to gauge how much useful information can be gleaned from them. Another limitation that could affect our analysis is that the internet is becoming increasingly widespread, embracing an increasingly wide range of populations. Now, you can access the internet with different devices, such as smartphones.

## Methods

Data from Google Trends is expressed as relative search volume (RSV). The RSV is presented on a standardized scale from 0 (no search interest) to 100 (greatest search interest). We have searched worldwide RSV for the English terms “gut microbiome, “dysbiosis”, “NAFLD” and “gut probiotics”. The search was limited to the period from 1 January 2007 to 31 December 2022. Data related to the public interest was downloaded in “csv” format from the Google Trends website^34^ on January 18, 2023. Since the data fluctuates over time, we have processed the data, which is shown as an annual average of the monthly values of RSV from 1 January 2007 to 31 December 2022. Our analysis used the country option to determine the geographical distribution of RSV for each searched term. Google Trends can also identify searches that are connected to a given search term and categorize these searches as “queries.” The top searches option displays popular search phrases that are comparable to the keyword input, during a specific time period. We analyze the 'top' searches for each term “gut microbiome, “dysbiosis”, “NAFLD” and “gut probiotics”.

## Data Availability

The data sets generated and/or analyzed during the current study are available upon request from the corre- sponding author.
